# The Works of John Hunter

**Published:** 1839-04

**Authors:** 


### Art. V.

1. *The Works of* John Hunter, f.r.s.; *with Notes by* James F. Palmer. In four Volumes. Vol. III. *A Treatise on the Blood, Inflammation, and Gunshot Wounds.—London*, 1837. 8vo. pp. 586.
2. *A Treatise on Inflammation.* By James Macartney, m.d. f.r.s. f.l.s. m.r.i.a. &c. &c.—*London*, 1838. 4to. pp. 214.
3. *Illustrations of the Elementary Forms of Disease.* By Robert Carswell, m.d., Professor of Pathological Anatomy in University College, London, &c. Fasciculus XII. *Inflammation.* — *London*, 1838. Folio. pp. 8.
4. *Teoria della Flogosi, di* Giovanni Rasori.—*Livorno*, 1837. 8vo. pp. 260.

*Theory of Inflammation.* By J. Rasori.—*Leghorn*, 1837.

The subject of inflammation is one of so comprehensive a nature, and capable of being treated in such a variety of ways, that our readers will not be surprised to learn that, of the four works dedicated to it, whose titles head this article, scarcely any two have more than a name in common. It will therefore be necessary to pass them separately in review; but we hope to be able to do this in such a manner as to maintain the unity of the subject, and to present to our readers an outline of the most generally approved and, in our judgment, the most philosophical opinions on the principal topics under discussion. We shall commence by giving a sketch of the scope and character of each of the treatises before us; and the proud eminence which the first has so long maintained as the highest authority on the scientific part of the subject demands an especial notice from us,—a notice which we are the more willing to give, because we are convinced that many of the present generation of our professional brethren are content to become acquainted with the doctrines of Hunter at secondhand, and thus receive them in a mutilated and distorted form. The present elegant edition of this incomparable work (we speak of it, of course, in reference to the state of knowledge at the time it was composed,) will afford to all an easy means of access to this last literary production of a great and gifted mind. It is one which contains vast treasures of thought, only to be acquired, however, by deep and laborious research: it is not, therefore, so much a work for the youthful student as for him who is already considerably advanced in professional knowledge. The notes and commentaries added by Mr. Palmer are for the most part of a very excellent character, and, in the first or theoretical portion of the treatise especially, give a fair view of the present state of knowledge on several interesting points, and place many of Hunter's opinions in a clearer light than that in which the text presents them. We cannot extend our praise, however, to all of those on the practical portion of the treatise; and we shall feel it our duty hereafter to advert to what we deem important errors in Mr. P.'s views of treatment. These, however, are so completely separated from the original text that they leave its value quite unimpaired.

A peculiar interest attaches to all Hunter's writings. He has left a name which must endure as long as any reverence for the great of past times remains amongst us. Such men give a spring and life to a community by their presence, their society, and fame; and we read their works with a delight which increases with the importance of the subject. The Treatise on the Blood, Inflammation, and Gunshot Wounds has been known to the professional public for nearly half a century. It was compiled, we are told by its author in his introduction, "from notes and memorandums of observations made in the course of twelve years' residence in London," and afterwards arranged while he was acting as a surgeon on the staff in 1762, during the expedition against Belleisle. From this period Mr. Hunter kept his manuscript beside him, making such corrections and alterations as further experience and observation suggested, till 1793, when the liberty taken by others with his discoveries made him determine on its publication, "both from a wish to preserve his own right, and also to give in a more perfect form what was thought worthy of the public even in a mutilated shape." Discreditable as this circumstance is to those engaged in such a transaction, it is nevertheless to be viewed as a fortunate occurrence, since it urged on Hunter (always jealous of his own claims) to undertake a labour which might have been otherwise neglected. Indeed, we doubt whether a man ever brings his faculties to bear with their full force on a subject, until he writes upon it for the instruction of others. To place it clearly before others, he feels the necessity of viewing it more vividly himself. By attempting to secure his thoughts, and fix them in an enduring form, he finds them vague and unsatisfactory, to a degree which he did not suspect; and toils for a precision and harmony of views, of which he never before felt the need. He places his subject in new lights; submits it to a searching analysis; compares and connects with it his various knowledge; seeks for it new illustrations and analogies; weighs objections; and, through these processes, often arrives at higher truths than he first aimed to illustrate. It is one of the distinctions of a great mind that it is prone to rush into twilight regions, and to catch faint glimmerings of distant and unbounded prospects; and nothing, perhaps, aids it more to pierce the shadows which surround it than the labour to unfold to other minds the indistinct conceptions which have dawned on its own. Unfortunately, only a small part of the treatise on Inflammation was printed, when Hunter was hurried away from his labours. In so far, therefore, it may be looked upon as a posthumous publication; a circumstance which would disarm criticism of its keener weapons, even if the length of time it has been before the public did not render it unnecessary for us to enter upon a full consideration of its varied contents.

One of the greatest objections expressed to Hunter's writings, and especially to the volume before us, is the difficulty and obscurity of the style; by which the mind, it is said, is wearied and exhausted, and too often no other recompense is yielded than a confused and indistinct perception of the subject of which he is treating. Much of this obscurity in the present case, Mr. Palmer informs us in his preface, is to be ascribed to the peculiar circumstances under which the work was given to the world.

"It was published under circumstances of haste, and at a period of the author's life when he regarded his state of health as very precarious, and when his faculties, oppressed by repeated illnesses, cannot be considered to have been in their full vigour: in addition to which, only a small part of the work was through the press at the time of the author's death, and therefore it could not receive the full benefit of his corrections, or even of those of his executors, who, in consequence of the numerous professional calls upon their time, were compelled to relinquish their task. Many errors, consequently, have been allowed to pass unobserved, both in the text and punctuation, which often vitiate the sense, and still more frequently render the meaning of the author extremely difficult of extraction." (p. xix.)

On this point we shall take the liberty of stating our own sentiments. We cannot admit that the reasons which have been assigned offer a just explanation of Hunter's obscurity of style: first, because they would only account for some inaccuracies of expression, or statements too hastily or insufficiently put together; and, secondly, because we find the same thing pervading his other writings, which were the productions of his cooler moments, and when he was comparatively free from those embarrassments which crowded so thickly around him during the last years of his existence. It appears to us that this defect—for, in any case, it must be so regarded—was incidental to and inseparable from his character, arising out of his habits of thought and observation, and his previously defective literary education. In truth, he himself was quite aware of it; for we find him stating to a friend, respecting his treatise on the Venereal Disease, that, in order to render the language intelligible, he met a committee of three gentlemen, to whose correction every page was submitted. While we mean not, therefore, to deny that the charge which has been made has some grounds, we think, at the same time, that it has been much exaggerated: and when we reflect that the obscurity of Hunter's style has deterred many from availing themselves of his invaluable labours, we cannot but regret the fastidiousness and effeminacy of modern readers. We are aware that simplicity and perspicuity are essential attributes of a good style; but there are others, as energy and depth of thought, equally noble and important; and in these we will not admit that Hunter has ever been surpassed. To be universally intelligible is not the highest merit. The best style is not that which puts the reader in the shortest time in possession of the author's naked thoughts, but that which is the truest image of a great intellect, and which conveys fully and carries farthest into other minds the conceptions and feelings of a profound and lofty spirit. A great mind, such as Hunter possessed, cannot, without injurious constraint, lower itself to the grasp of ordinary individuals. Its own natural movement is free, bold, and majestic; and it ought not to be compelled to part with these attributes in order that the multitude may be able to keep pace with it. For our own part, we prefer easy reading, especially in our moments of relaxation; but we delight, at the same time, in having our faculties tasked by master spirits. There are many writings which are clear through their shallowness. There are minds, again, which grasp at once vast fields of thought, just as the eye surveys at once wide fields of grandeur and beauty; and which, in their moments of inspiration, when thick-coming thoughts and images crowd in upon them, pour out their treasures in a manner perplexing to ordinary readers, but kindling to congenial spirits like their own. We would not have all compositions of this character, but we would impose no over-strict laws on a great mind. We would let it address us in its own language, and in a tone which accords with its own ear. If not understood at the time, let it look forward with a generous confidence to the improvements of succeeding ages, and utter thoughts which others in future years will unravel.

We have been led to make these remarks, not so much in vindication of Hunter as that we think our own times seem to demand them. Medical literature is becoming in some respects too common and too popular: the whole community is now turned into medical readers. With this we so far acquiesce; nay, we rejoice that so much talent has been employed in making a certain kind of knowledge accessible unto all: we look, indeed, on the general diffusion of knowledge as one of the noblest and most distinguished features by which the coming will be separated from past ages. But good is often conjoined with evil; and we fear lest men of genius be led away, by the shout of popular applause or the desire of obtaining sudden wealth, from pursuing that deeper and stiller path where that thrilling note alone is heard which sounds louder and clearer throughout all succeeding generations.

The treatise on the Blood and Inflammation has been regarded as the greatest effort of Hunter's genius, and that upon which his reputation has principally rested. We do not assent to this opinion: it is only from those unacquainted with his other works that such an opinion could have escaped. Without meaning to disparage it, we may say that it owes very much of the attention which it has excited to the previous fame of its author, and to its having been the first in which the doctrines of inflammation were set forth in a luminous and philosophical manner; but, when compared with Hunter's stupendous manual labours, it sinks far into the shade. We value it chiefly as showing us the mind of a master on a subject which, above all others, presses itself upon the attention of the physician or surgeon, both from the frequency of its occurrence and its intimate connexion with diseased action. We are desirous of ascertaining in what conclusions such a man rested, after a life of magnanimous efforts to unfold the hidden laws of nature, and to trace the order and regularity with which they occur. The work before us shows that Hunter's natural progress was from one field of discovery to another; that he could give a freshness and vividness to truths which had become worn, we had almost said tarnished, by long and familiar handling; that he could bind together, by mysterious affinities, remote discoveries, and rear fabrics of utility and beauty from the rude materials which other minds had neglected. His intellect seems to have been excited and developed very much by the circumstances under which he might chance to be placed; it was naturally creative, restless, stirred by a burning desire for the discovery of truth, and he was conscious of that within him which could quicken all knowledge and wield it with matchless power. In treating of practical subjects, he had not learned the superficial doctrine of later days, of discarding or setting at nought legitimate theory; his object being to combine the two, and make the latter arise naturally out of the former. His published lectures make us best acquainted with the importance which he attached to the attainment of fixed principles; for, as he himself has justly remarked, "without such knowledge a man cannot be a surgeon." If, indeed, there is an error to be found in the present treatise, independent of those which the subsequent progress of medical science has developed, it is perhaps the undue preference assigned to this sort of investigation, and the attempt sometimes made to compel medical treatment to yield to it, however much at variance in many cases with the plain and obvious dictates furnished by experience.

The profound and searching views contained in this treatise demonstrate, in every page, Hunter's attachment to reasoning. A mere discovery, or the knowledge of merely a practical fact, was to him unsatisfactory unless he could bring his mind to comprehend the successive changes as they arose. This is another circumstance which has rendered his writings so valuable; and if he has frequently failed in following out the true process of reasoning, or in referring effects to their true causes, we must still admire the unextinguishable ardour with which he pursued the investigation. We by no means desire to set up theoretical in opposition to practical knowledge; and still less to form rules of treatment on the one which are irreconcilable with the other. We would rather, indeed, that the order were reversed. What we would wish is, that the two should, as far as possible, go hand in hand, in order to infuse a true and philosophical spirit into the study of medicine. The individual who cultivates either separately and independently retards rather than accelerates his own science. In the one case, at the bedside of a patient, he only appears as a sort of learned spectator; in the other, we regard him as little better than an acting automaton. The history of the medical literature of our own and of other countries confirms the truth of these statements. If, in former years, too much time was spent in purely speculative enquiries, in the present day the same error has been committed in the cultivation of practical knowledge. No real advancement can be made without the acquirement of sound principles, arising out of and confirmed by daily experience. They are to the medical man what the rudder is to the mariner. Their disjunction cannot be sufficiently condemned. Such a system has long enough warred against the interests of the human race, and arrested the progress of improvement. The time for its fall, we trust, is coming : it cannot come too soon.

We prize, then, the volume before us, as being the first in which more just views were disseminated, and a new light thrown over the subjects of which it treats. Previous to its appearance, the study of inflammation had been little attended to, and many of the phenomena which accompany it were ill understood. "I have endeavoured," remarks Mr. Hunter, in his introduction, "as far as my other pursuits would permit, to form this work into a regular system, one part exactly depending on another. How far I have succeeded the world must judge. But, at the same time, it ought to be considered *as a new figure composed from rough materials,* in which process little or no assistance could be had from any quarter; wherein the author is conscious of many imperfections, more of which, he is persuaded, he shall himself observe at every successive review."

Mr. Hunter may be considered the first who classified and arranged the various phenomena of inflammation; and so accurate are many of his illustrations that they have not only remained undisturbed, but little, if any, real addition has been made to them up to the present period. To say that the work is without errors, would be to say that its author was not a man. False opinions are the rank growth of a soil which requires to be weeded as well as tilled; and we have doubtless to regret that Hunter did not live to correct those imperfections which are to be found in some of his pages. At the time the treatise was commenced, animal chemistry in particular was in a rude and imperfect state; the minute analysis of the blood was unknown; and all the phenomena which depended for explanation on chemical science were necessarily defectively detailed. Hence the reason why so many corrections are required in the new edition, to bring down this portion of the subject in accordance with the more correct views of the present day. This, however, was not the fault of Hunter, but of the age in which he lived. In some of the other departments, too, we shall have occasion to show that his views were occasionally erroneous; but at this we need not express surprise, seeing that the ground had been previously untrodden. We do not expect, in any work which has for its object the investigation of diseased action, to meet with statements which shall always remain uncontradicted, or inductions which the progress made in future years will not show to have been hastily drawn; but in Hunter's writings we find less of this than might otherwise be anticipated: his views of the animal economy, either in health or disease, were too profound, and culled from too imperishable a source, not to be in the majority of instances correct.

It has also been made a matter of complaint that Hunter should have been so little acquainted with the labours of his predecessors, or even with those of his contemporaries in his own and other countries. On this account he was himself frequently surprised that many of his discoveries had been anticipated, and the student is often at a loss to know to whom the original merit of these is due. The wisdom of every age, we may remark, is chiefly a derivation from all preceding ages, not excepting perhaps the most ancient, and should hold communication with them, just as a river, through its whole extent and in its widest overflowings, still continues to receive tribute from its infant springs. We wish not, therefore, to acquit Hunter of culpable neglect in this respect; although in his case we regret it the less, because it made him draw more deeply from the full fountains of his own vast and comprehensive mind. By his researches into the book of nature, he rendered to his profession a far greater service than if he had moulded into new and more beauteous forms the wisdom and the wealth of all his predecessors. He taught and exemplified that spirit of intellectual energy through which all the great conquests of truth have been and are to be achieved.

We have been induced to make these general remarks to save us the necessity of extracting largely from a work which has been so long known to the profession. Had the case even been otherwise, and the volume for the first time before the public, we should have entered most fully into the sentiments expressed by Mr. Palmer, in his preface, "that those who would apply the ordinary rules of a petty criticism to the present work will form a very incorrect judgment of its merits; while those who come to its perusal in the expectation of finding a simple exposition of disease will probably be much disappointed. The reader who would form a just and accurate appreciation of a work of this character should bring to the task considerable liberality of mind, and at the same time an independency of judgment, which can admit the general propriety of an observation, and yet know how to make those due and necessary abatements which all general propositions require." We would, then, recommend this volume, with the other writings of Hunter, to all who feel any interest in the study of their profession, or who have any desire to become acquainted with the labours of a master-spirit. There is no calculating the good their diffusion may effect, if it were only by creating in the breasts of others the love of those pursuits—all ministering to the good of man—amid which he lived and died. Far, however, from regarding Hunter as standing alone and unapproachable, we believe that he is an illustration of what many who dream not of this might arrive at in the course of their being. In his unwearied and unexampled industry and perseverance we find the germ of all his greatness; and we would hold out his fame, not to excite an ineffectual admiration, but to awaken others and ourselves to the free use and expansion of our noblest faculties.

Hunter's treatise opens with a few introductory remarks, 1st, on diseased actions, as being incompatible with one another; 2d, of parts susceptible of particular diseases; 3d, of sympathy; 4th, of mortification. The three former will be found more fully illustrated in his Lectures on Surgery. It is not a little remarkable that the latter should be so briefly treated of in this place, and that the consideration of it as a consequence of inflammation should have been altogether left out of view in the body of the work. Had Mr. Hunter lived, we have little doubt that he would have seen the propriety of remedying this omission; and we should have been better pleased had the defect been supplied in the present edition by a few notes in the shape of a commentary. We shall make a few remarks on Hunter's views on the nature of mortification, when treating of it as a consequence of inflammation, from which this process undoubtedly results in the majority of cases.

We shall take another opportunity of reviewing the first part of Hunter's treatise, which consists of the chapters on the Blood and Vascular System: on the present occasion we shall confine our attention to the second part, devoted to the subject of Inflammation. Of this, the first chapter is set apart for a subject that cannot be legitimately considered under this head, namely, Union by the First Intention, of which we shall say more hereafter. The following titles of the remaining chapters of this part will give a general idea of the nature of the author's plan. ii. Fundamental Principles of Inflammation. iii. The Adhesive Inflammation. iv. Of the Suppurative Inflammation. v. Of Pus. vi. The Ulcerative Inflammation. vii. Granulations. viii. Of Skinning. ix. Effects of Inflammation, and its Consequences on the Constitution. Many of these subjects we shall touch upon, in what appears to us their natural order, in the present or in a future article; and we shall here only remark that, under the title of *adhesive inflammation*, Hunter does not merely comprehend, as many in the present day might be apt to imagine, the phenomena which accompany the process of adhesion, but what we consider and speak of as *common inflammation;* that is, inflammation not as yet followed by any ulterior effect, as suppuration or ulceration. Hence in this chapter we find him discussing the state of the vessels in inflammation, the local and constitutional signs of the disorder, the effects on the system according to the structure of the part affected, and the remedies necessary for its removal. One of the most interesting points of enquiry, and which has received a large share of attention and been made a subject of keen discussion, is the actual state of the vessels of an inflamed part. Considering the amount of labour and time spent in this investigation, we are naturally curious to know, even at this distant period of time, in what conclusions such an acute observer as Hunter rested. It does not appear, however, from anything which we can detect, that he had ever examined the process of inflammation experimentally, or as it is seen going on in the transparent parts of an animal texture, and he seems to have remained contented with theorising on the subject. An examination of allusions which he makes to it will show that, while some of his notions were correct, he was at the same time drawn into inconsistent statements, and that he evidently experienced, as might be expected, the greatest difficulty in making up his mind on this abstruse question. The remainder of the treatise, consisting of Part III. on the Treatment of Abscesses, and Part IV. on Gunshot Wounds, does not call for any present remark from us.

Of the general character of Dr. Macartney's treatise we have already stated our opinion at p. 221 of the present volume; and, in pursuance of the plan with which we set out, we shall explain the objects and qualities of the two other works on our list, before proceeding to examine in detail the doctrines set forth in them. That of Dr. Carswell consists principally of details as to those changes of structure produced by inflammation, which it is the object of his very beautiful plates to represent; but he has prefaced them by an account of his opinions as to the proximate cause or essential nature of the disease; and upon this we shall offer some remarks at a future period. The "Teoria della Flogosi" of Rasori cannot, however, be so summarily dismissed; and, that our readers may enter fairly upon the consideration of a work which is by many regarded as on a par with that of Hunter, we shall preface our account of it with a short exposition of the principles of the school from which it originated.

To the new Italian medical school our profession stands largely indebted for its present augmented and more correct knowledge of the precise degree of influence exercised by many powerful agents on the animal economy, as well as of the condition of body by which their effects are modified, and the extreme limits within which their employment is at once most efficacious and safe. Its founders complained bitterly of the manner in which its tenets were misrepresented on their first introduction to the public. Thus, by M. Broussais, one of the most conspicuous of the writers who have undertaken to criticise them, their system was brought under the notice of the French, some twenty years ago, under the injurious and incorrect appellation of "Italian Brownism." Tommasini, one of the most celebrated of the expositors of the new Italian doctrine, whilst he admits its being founded in part on the first principle of Brown's theory,—namely, the relation of organized bodies to stimuli,—asserts that in its superstructure, whether considered theoretically or in regard to its leading practical precepts, it differs totally from the system of the Scottish innovator. Thus, the almost universal stimulant nature of external agents, and the doctrine which views indirect debility as the cause or essence of almost all diseases, are both emphatically denied by the Italian reformers. Their doctrine of contra-stimulus is in direct opposition to the first of these dogmata; and their assertion that diseases originating in excess of stimulus preponderate mostly over those of an asthenic character, is equally at variance with the latter. Amongst the disciples of Rasori, Tommasini has especially occupied himself in adducing proof of the sthenic nature of all inflammations.

As so much error has prevailed in regard to Rasori's real opinions, and as the doctrine of contra-stimulus tinctures nearly every page of his work, it may be well briefly to enumerate its leading tenets; these are as follows: 1st. There are a great variety of substances, such as antimony, mercury, &c., which produce on the organization an effect diametrically the reverse of stimulant; controlling excitement in a direct manner, and producing those consequences immediately which were erroneously looked upon by Brown as secondary, or as the product of negative causes, i. e. of the mere diminution of stimulants. 2d. These substances, comprised under the general term contra-stimulants, often manifest their highest influence where they give rise to no increase of the evacuations. 3d. If employed unnecessarily, or pushed too far, they produce a new morbid state, which can only be subdued by the exhibition of stimulants. 4th. The magnitude of the dose of stimulants, or of counter-stimulants, which can be borne with impunity in any case, is exactly in proportion to the degree in which the opposite diathesis of counter-stimulus, or stimulus, (terms nearly equivalent to diminution or increase of vital action,) prevailed. 5th and lastly. This tolerance of medicinal agents, experimentally ascertained, affords us a much surer index and measure of the diathesis we have to deal with than do the symptoms. Such were the opinions promulgated by Rasori in the latter end of the last century, and his belief in them only ended with his life.

The present work on Inflammation, the last of Rasori's publications, is, we are told, the fruits of forty years' careful study of the phenomena of diseases and devoted attention to morbid anatomy. The collecting of materials for it, though occasionally interrupted by the disturbed state of affairs in his unhappy country, alternately the theatre of foreign war and of domestic revolution, was never, even under the most adverse circumstances, long lost sight of. Conceiving that the inductive mode of prosecuting medicine has never yet been fully adopted, and that, till this is the case, there can be no hope of its being fitted to take its station fairly by the side of the other sciences, his aim in the present treatise is to set an example, which, if followed up in the same spirit, may soon lead to the removal of this reproach. Useful induction presupposes the possession of an adequate number and variety of well-observed and carefully-recorded facts: of these Rasori is persuaded there is still a deficiency, and to assist in supplying it he states to be his first object.

In his attempts at ascertaining wherein the essence of inflammation consists, he believes himself to have taken an opposite course to that ordinarily pursued. "Others," says he, "commence their investigations by examining inflammation as it constitutes a morbid state of function in the living; but I begin, on the contrary, by considering its effects such as they remain in the dead body." Whatever novelty there was in this method when Rasori first entered on it has long ceased to exist, the

French school having made morbid anatomy the basis of nearly all their reasonings about disease, and represented its importance, if possible, even in an exaggerated point of view.

We shall notice the principal peculiarities in Rasori's work in their proper order; but we cannot help feeling that, characterized as it is by an inordinate love of theory, a dogmatical assertion of dubious facts, and a frequent feebleness of argument, neither medical science nor the character of its venerable author would have suffered materially though it had never seen the light. Compared with Hunter's treatise, rich to overflowing in original experiments, careful inductions, and profound philosophical views, the feebleness and poverty of the Italian professor make themselves painfully felt. How much he is in arrear of the present state of pathological knowledge may be deduced from his denying the possibility of an unnaturally large heart having any concern in the production of a coexisting dropsical affection; as well from his making no allusion to the condition of the interior of this organ, its valves and orifices, and lining membrane, in cases of hydropericardium, &c. As to his practice, we feel that we should not be doing our duty towards the junior members of our profession, were we not to caution them strongly against its rash adoption in acute cases; and remind them of the fearful habits of intemperance in the use of opium and vinous stimulants which could not fail to result from its extensive and unguarded application in chronic disease.

It is a prominent feature in Dr. Macartney's work, and one in which it differs from all treatises on the subject, that the author regards the *reparatory* and *protective* processes as essentially *non-inflammatory*. Of this character he considers not only union by the first intention and by granulation as partaking, but also the effusion of plastic lymph under any circumstances. He also directs attention to a mode of reparation which he terms the *modelling* process, or reparation by *natural growth*, as distinct from that by granulations. Although the peculiar effects of this process, occurring under certain conditions, are by no means unknown, as we shall presently show, to practical men, yet we believe this to be the first time that the *rationale* of it has been displayed, and the conditions on which it depends satisfactorily demonstrated. As we are disposed to coincide in Dr. Macartney's views on these subjects to a considerable extent, we shall give them in his own words, with some abridgment.

In the first place, he points out that the reparative processes cannot be as dependent on inflammation as a superficial observation of them in the human subject has induced pathologists to believe: since, in the lowest tribes of animals, and in plants, in which these processes are carried on to their greatest extent, there is no such thing as inflammation. We are not aware that this distinction has ever been made before; and we would recommend to our readers the perusal of Dr. M.'s first chapter on the History of Inflammation, as an admirable specimen of the advantage of the study of comparative physiology to the pathologist, whose attention is directed to the effects of disease in man. It is well known that, in the inferior tribes of animals, as in plants, the powers of reparation exist to an extent, of which their effects in man can be regarded but, as it were, the remnant. In proportion as the different parts of the structure become more similar to one another, as in the articulated and radiated classes, does their dependence on each other become less; and in that most extraordinary creature, the hydra, or green polype, in which every part of the fabric seems but a repetition of the rest, multiplication of individuals by artificial division of one may be practised to almost an indefinite extent; the separated portions, not only of the body, but even of the tentacula, gradually developing themselves into perfect hydræ. Now, if reparation takes place in such instances as these by a process of natural growth, in which inflammation is not at all concerned, we think it a fair argument that the same should be looked for in man; though an argument of *analogy* only is not one on which we are yet entitled to rely in physiology, if facts closely examined are in opposition to it. But we shall find reason to agree with Dr. M. that the weight of these facts is really on his side; and in this instance, too, the analogy is really closer than might be supposed by a superficial observer; for, although the multiplication of individuals by the division, whether natural or artificial, of the original structure might seem to have nothing to do with the reparation of injured parts in the highest tribes, a little observation will show that the processes are essentially the same. For, in the polypes, and others of the inferior tribes, we find every part of the structure capable of becoming a new individual. In the radiata and higher articulata, there is very considerable power of reparation of lost parts; the star-fish, for example, acquiring new arms, and the crab or spider new claws; but these separated parts are not capable of becoming new individuals. In the cold-blooded vertebrata, we still find a remarkable power of regenerating members, that, once lost in birds and mammalia, are seldom or never replaced: but, even in the latter, we find a capability of reproducing parts of the fabric that have been destroyed by disease or injury, really no less remarkable than that enjoyed by the lowest animals, since the processes concerned in it are of a still more complex nature: one of the most extraordinary of these is the reformation of a necrosed bone, and its gradual restoration from a shapeless mass to its perfect form. The more closely the analogy is enquired into, the more perfect, we are convinced, it will appear to be. To these general observations we shall subjoin a few sentences from Dr. Macartney's chapter on the subject.

"There is no reason for believing that a process of the same nature on inflammation exists under any circumstances in the vegetable kingdom. When *plants* are injured, or have a part of their substance destroyed, there is no new process of reparation set up, but the vacancy is filled by the regular and natural growth of the vegetable body. . . Animals that have no visible nerves, and those in whom the nervous system is very simple, exhibit none of the phenomena of inflammation . . . When *worms* are cut into two portions, the divided surfaces assume the disposition to heal from the moment the injury is inflicted. The edges of the wound approximate each other, and remain in close contact until they are perfectly healed, after which they recede to restore the tubular form of the animal. . . It does not appear that *insects* are susceptible of inflammation after injuries. It is not known in what manner the poison generated by certain species of insects affects others of the same class; but it is quite certain that it does not cause inflammation as in quadrupeds and the human subject. When the spider inflicts a wound on a fly, it is instantaneously fatal; but when a sufficient quantity of the poison is not inserted into the wound, it stupifies the insect, producing the effect of a vegetable narcotic poison.* . . . The class *mollusca* do not seem to be capable of genuine inflammation. In some of the *testaceous mollusca*, as the *oyster* and *muscle*, we find that some parts of the body may die and putrefy, yet remain in connexion with the rest, and apparently not affect it. The presence of an extraneous body will produce an exuberant growth of the shell, causing a greater flow of the juice of which the shell is composed; and thus the growth of pearls is perfectly similar to the excrescence of plants caused by the gall insect. . . . In neither of the two classes of vertebrate animals with cold blood, do I believe it is possible to produce the genuine effects of inflammation. In conducting some experiments on the swimming bag of *fishes*, I was surprised to find that the wounds made into the belly of the animals did not inflame. I was therefore curious to know what injuries fishes would bear without producing inflammation. Having taken some living fishes from the water, I introduced pieces of wire beneath the skin and amongst the muscles of the body; the fishes were then returned to the water, and on examining them several days afterwards, I found that no suppuration had taken place. The tracts of the wounds were pale and smooth, and only moistened with a serous fluid, and none of the usual appearances of inflammation were visible. A very common occurrence in fishes is the existence of worms, which perforate the tunics of the alimentary canal, without producing any change of structure, except an increased vascularity around the perforations. . . . I have never seen any appearance of inflammation in *reptiles* after wounds or injuries. *Serpents* often lose a portion of their tail; and although there is no attempt made for its reproduction, it is very speedily cicatrised without inflammation. . . . In the class of *birds* we first discover the existence of genuine inflammation as the consequence of external mechanic injury; but the instances in which internal disorders become a cause of inflammation, are very limited, and are nearly confined to febrile states, and particular epidemics. *Quadrupeds* are subject to inflammation, both from external injury and internal disorders; they usually, however, show but little constitutional sympathy with local disease. A dog or a horse will continue to eat, although suffering from an accident or a disease that may prove fatal. The human being, above all others, is disposed to inflammation; sometimes in consequence of the slightest external irritation, and of various internal disorders. The nervous system of the human subject is so complicated, that there is hardly a local affection with which the constitution does not sympathise, nor any constitutional disturbance which may not become the cause of local disease. The same susceptibility, however, communicates a power to the means we may employ for preventing or abating inflammatory action, which does not belong to animals of inferior organization; and when by these means we are enabled to remove the sense of injury sustained, or produce a state of sensibility inconsistent with inflammation, the reparative processes go on much in the same manner as in animals endowed with an inferior degree of feeling." (pp. 1-6.)

From the class of facts of which we have thus given an outline, Dr. Macartney draws the following inferences:

"That the powers of reparation and of reproduction are in proportion to the indisposition or incapacity for inflammation; that inflammation is so far from being necessary to the reparation of parts, that, in proportion as it exists, the latter is impeded, retarded, or prevented; that, when inflammation does not exist, the reparative power is equivalent to the original tendency to produce and maintain organic form and structure; and that it then becomes a natural function, like the growth of the individual or the reproduction of the species." (pp. 6, 7.)

* To this we may add, that the bee's sting seems to have a similar effect upon individuals of its own species. This is exemplified in the annual massacre of the drones, and in the contest which often takes place between two queens: in the latter case, the whole efforts of each combatant appears directed towards the insertion of the sting in the body of the other.—REV.

Agreeing, as we do, with Dr. Macartney in the inferences themselves, we must take leave to say that we do not regard the data on which they are *here* erected as sufficient to establish them. The mere difference between the state of the blood in warm and cold-blooded animals might, for anything we could *à priori* know to the contrary, create an essential variation in the character of the reparative processes in the two classes respectively. It will be only after we have ascertained, by experience, that these processes go on best in the former when the parts concerned are reduced to a condition resembling that in which they exist in the latter, that we shall have a right to say, that inflammation is in its very nature opposed to reparation. Another deficiency we must point out in Dr. M.'s argument, which has an essential bearing upon his *theory* of inflammation. Nearly all his statements are confined to the effects of mechanical injury; and although he may be quite right in saying that these are best repaired when inflammation is not excited by them, he has no right to assert, as he does, that inflammation never exists in vegetables or in the lowest animals. We are not, of course, to look in them for the same phenomena as those which present themselves in animals with warm blood and complex nervous system; but all vegetable physiologists are agreed, that phenomena corresponding with inflammation may be excited by certain irritants in the parts to which they are applied, or in distant organs if they are taken into the current of the circulation. This has been distinctly proved by the experiments of Drs. Turner and Christison on the effects of poisonous gases on vegetation;* and by those of MM. Macaire and Marcet on poisons applied in solution.† The irritant agents are found to have an effect quite opposite to that of narcotics, producing a temporary turgescence and rigidity of the parts, the vitality of which, however, is speedily destroyed by over excitement; whilst narcotics produce an immediate flaccidity and loss of irritability.

Dr. Macartney has very properly pointed out that Hunter, "in his inestimable work on inflammation, when describing the union by what is called the first intention, seemed aware of the possibility of wounds healing without any inflammatory action; he says, the union in such cases is without pain or constitutional disturbance, and 'proceeds as if nothing had happened;'" and that, in consequence of the apparent contradictions in different parts of his treatise, he "has been quoted as an authority for opinions directly at variance with those principles of physiology which it was the great object of his life to establish." Pathologists who consider themselves as belonging to the Hunterian school have assuredly substituted, in many instances, their own commentaries for the opinions of their master; and of this we shall presently adduce some striking illustrations. We shall now examine the doctrines put forth by Dr. Macartney on the reparation of injuries, and compare them with those of Hunter, and other pathologists at the present day. According to Dr. M.,

"Reunion and reorganization are effected in four different ways, which may be designated in the following manner:—*First*, immediate union without any intervening

* Edinb. Med. and Surg. Journ. Vol. 28.

† Ann. de Chimie, tom. xxix.; and Bibliothèque Universelle, tom. xxxi. See also Lindley's Botany, chap. xiii.

substance, such as blood or lymph. *Second,* the union by a medium of coagulable lymph or a clot of blood. *Third,* reorganization without any medium of lymph or granulations, the cavity of the wound becoming obliterated by a natural process of growth. *Fourth,* the reparation by means of a new, vascular, and organized substance, called granulations.

"To the first of these modes of cure, I should wish to give the name of *immediate.* The second may be called the *mediate by lymph or blood.* The third, being compounded of different actions, I find a difficulty in distinguishing it by a single name. It might be denominated the *approximating* or the *modelling* process of reparation, or that by a *natural growth.* The fourth mode of union should be termed *mediate by granulation.* The first three modes of restoration are quite incompatible with the presence of inflammation; a slight degree of which may, however, exist with the fourth." (p. 48.)

We confess to have our doubts how far the first mode of union, described by Dr. Macartney, can be legitimately regarded as distinct from the second. The following is his account of it:

"The circumstances under which immediate union is effected are the cases of incised wounds that admit of being, with safety and propriety, closely and immediately bound up. As no intermediate substance exists in a wound so healed, no mark or cicatrix is left behind. We have familiar examples of this mode of healing, in slight cuts received on the fingers, which, after being bound up, if no inflammation be induced, perfectly heal without the individual having any unpleasant sensation in the part, after the moment of the infliction of the wound." (p. 49.)

Now we cannot understand how union can take place between two divided surfaces without the medium of some solidified effusion, however small in quantity it may be. Although the illustration we shall employ is a rough one, yet it will, we think, have the merit of fidelity. The artisan who employs glue to unite two surfaces is perfectly well aware that the thinner the film of glue which remains when they are applied, the more firmly will his work hold together, the sooner it will set, and the better it will look. Now the solidification of the glue in this case answers precisely the same purpose, as far as mechanical union is concerned, with the organization of coagulable lymph; and it appears to us that, where the two surfaces admit of being brought into close apposition, the film effused between them will necessarily be of extreme tenuity; its entire organization will take place with proportional rapidity; and the traces of it will disappear most completely. Without such a film we cannot conceive that any union can take place; and Dr. Macartney does not offer any evidence, saving the rapidity of the union, and the absence of a cicatrix, against the usually-received doctrine of its formation. We have known, too, instances in which incisions that had apparently healed completely, in the manner here alluded to, burst asunder three or four days afterwards through an accidental tension of their edges; and it is evident that no organic union could have then taken place, the thin film of lymph still acting only as a glue.

The second mode of union is described by our author as follows:

"The union of parts with the medium of lymph or blood takes place in wounds which either cannot, from the extent or shape of their surfaces, be brought into perfectly close contact, or where the parts will not sustain much pressure, without the danger of inducing inflammation. The lymph which issues from the adjacent surfaces, in the first instance, glues them together, and in a few days is found to have acquired some vascularity, and an imperfect degree of organization; after which an external restraint for keeping the divided vessels in contact becomes unnecessary." (p. 50.)

Of this kind of union, the healing of the puncture made in venesection may be taken as a simple example. This may be healed with scarcely any intervening lymph, and with no consciousness on the part of the patient; but it leaves a line of white cicatrix, because it would not be justifiable to apply in this case the same degree of pressure as in the small cuts of the fingers, for the purpose of retaining the edges of the wound in close contact. According to the common opinion of British surgeons, an *adhesive inflammation* is required to effect this union; and the following passage, quoted by Dr. Macartney from Sir A. Cooper's Lectures on Surgery, may be regarded as an *ex cathedrâ* expression of this opinion in its broadest form. "Inflammation is a restorative process; no wound can be repaired without it; even the little puncture made by the lancet in bleeding would inevitably destroy life, if this salutary process did not prevent it." This, as we shall presently see, is an assertion which the teachings of Hunter by no means warrant. "I am well pleased," says Dr. M., "that the doctrine has been thus so clearly and unequivocally asserted, that no doubt can exist respecting the meaning of the author." The following are his general remarks upon it:

"Every one knows that, if the puncture of venesection be provoked to inflame, or in common language to fester, its progress towards healing is interrupted; yet this very wound has been quoted by Sir A. Cooper as an example of one requiring the aid of inflammation for its reparation. Those who contend for the necessity of inflammation in all cases for the reparation of injury cannot refuse to admit that, in the instances I have just mentioned, none of the phenomena ascribed to inflammation exist; and that whenever the surgeon intends to accomplish union by the first intention, his success will depend on his being able to keep the parts in so easy and tranquil a state, that none of the phenomena of inflammation make their appearance. If there be any degree of inflammation, in which there are no heat, redness, tumour, pain, or disturbed vascular action, it ought to be clearly distinguished from that kind which is attended with these phenomena, and then we should have two sorts of inflammation; the one with phenomena, the other without, which, if we chose to disregard the logical contradiction involved in such an admission, would amount to the same practical result as if on one occasion inflammation did exist, and on others did not." (p. 50.)

"Every surgeon knows that, if wounds united by means of lymph be provoked to inflame by motion, friction, too much constraint, or any other cause of disturbance, the tender and half-organized medium is destroyed, and the chance of union by the first intention is lost. We cannot, therefore, with any consistency, describe the adhesive process as an inflammatory one, when we perceive that it is interfered with by any sensible degree of inflammation." (p. 52.)

"It is well known that coagulable lymph may be thrown out by a natural and healthy action, as in the formation of the decidua uteri; that it is eminently conservative, in arresting hemorrhage from opened vessels; in the union of soft parts when divided; in forming the medium of conjunction of fractured bones, and in constructing the walls of an abscess and of an aneurismal sac. Immediately on the receipt of an injury, also, the lymph is shed before there is time for inflammation to set in. The surface of a wound that does not bleed is covered with a layer of lymph, in the very moment that the injury is inflicted. The inflammation which would ensue from the opening of a serous sac is sometimes altogether averted, and almost always restrained within certain bounds by the effusion of lymph uniting the opposed surfaces with each other." (p. 38.)

With the greater part of these views we entirely coincide; and a careful comparison of them with the original doctrines of Hunter will show that there is more resemblance between them than most of the disciples of the latter would be willing to allow. In the treatise of the latter is a chapter of considerable length, which treats of *union by the first intention;* and we here find a full description of the mode and circumstances in which this takes place, and a distinct and careful separation of its phenomena from those of inflammation. The medium by which he regarded this union to take place is not essential to the question. It is well known that Hunter considered the fibrin of a clot of blood as capable of becoming organized, and as forming the connexion in the instance in question; and on this point most pathologists of the present day are at issue with him. Dr. Macartney seems to agree with him, however; as he states that he has seen vessels passing for a short way into a clot of blood covering the surface of an ulcer, when the coagulum possessed no vascularity of its own; and that he has succeeded in forcing injection into the coagula formed in the cavities of the heart after death, which presented the appearance of red elongated lines. These considerations also lead him to believe that blood-vessels never *originate* in effusions of blood or lymph, but are prolonged into them from the neighbouring tissues. On this question we have given our opinion elsewhere.

Surgeons of the present day, however, in abandoning the idea that an organized coagulum of blood ever becomes a medium of union, have altogether given up the notion of the true "union by the first intention," described by Hunter; and we accordingly find Mr. Palmer thus commenting upon the chapter in question:

"It is now generally considered that *union by the first intention* and *adhesive inflammation* are essentially the same processes, modified by the degree of inflammation. Union by the first intention is uniformly attended with some degree of pain and swelling, together with increased heat and vascularity, which, taken conjointly, constitute the definition of inflammation. . . . There seems not the slightest reason for believing that the mechanism essentially differs by which naturally-contiguous parts morbidly adhere, and that by which artificially-divided parts unite from a principle of conservation; and yet all the phenomena of adhesion in disease uniformly point to the presence of inflammation and the effusion of coagulable lymph." (*Palmer's edition of Hunter*, vol. iii. pp. 254, 268.)

We think that there is this essential difference—that inflammation is injurious to the process in one instance, and is the cause of it in the other. The first is a physiological, the second a pathological action. But we are anticipating another very important question raised by Dr. Macartney, to which we shall presently return.

The doctrine that the effusion of lymph for the reparation of the tissues is not to be regarded as an inflammatory process is by no means peculiar, however, to Dr. Macartney, nor does he claim for it any originality. It is in fact as old as the time of Galen.* John Bell maintains that the term *adhesive inflammation* is inapplicable to this process, which he regards as purely physiological. Maunoir, Delpech, Serres, Roche, and Sanson, and other pathologists of the physiological school, have adopted a corresponding opinion. Even Hunter, to use Mr. Costello's words,† "in his division of inflammation into adhesive and suppurative, seems less anxious about entering into a disquisition as to the constant presence of inflammation for the production of adhesion than to mark the phases of the vital act that produces it. The idea

* See Cyclop. of Surgery, vol. i. p. 49. † Op. cit. p. 50.

which he connects with this act is that of increased activity of the nervous and capillary systems, with augmentation of the plasticity, which, he is a witness, will often arise without pain, redness, heat, or swelling. The sense which he attaches to the term *adhesive inflammation* is that of an action in which the vessels become capable of secreting the plastic element; and in this Thomson, Cruveilhier, and almost all the pathologists of this country coincide." The question, then, as Dr. Macartney has already remarked in the passage quoted above, is more one of words than of facts; being, whether the term inflammation shall be used to denominate a state in which plastic lymph is thrown out, without sensible increase of heat, pain, or tumefaction; or whether it shall be restricted to conditions in which these manifest themselves. We certainly think, with him, that the precision, of which there is such a sad deficiency in medical language, imperatively requires the latter course, that confusion may be avoided. That increased vascularity cannot be regarded as of itself constituting inflammation is allowed on all sides; and if the natural process of the formation of the decidua uteri be regarded (which it usually is) as a non-inflammatory action, we see no great difficulty in extending the same principle to the reparative processes under discussion, which seem to us perfectly analogous. The human body, in its healthy condition, is possessed of the power of executing all the actions necessary for the maintenance of its own normal constitution, and for the production of new beings; and the processes which form a regular part of these can scarcely be regarded as abnormal. We have seen how closely in the lower animals the function of reproduction is connected with that of reparation; and that, in the highest classes, the degree of the latter power which is retained is to be regarded as a part only of the former. There is an essential distinction, then, both in character and in phenomena, between actions of this kind and the diseased conditions to which, at first sight, they assimilate.

"But," we can imagine many of our surgical brethren thus objecting to such a doctrine, "every practical man knows that there are many states of the constitution in which wounds will not unite, owing to deficiency of inflammation; we see their edges pale and flabby; and as soon as they become red and tumefied, union begins to take place." These facts, however, are susceptible of a somewhat different explanation. Every one knows that there are conditions of the system in which the blood, from various causes, is deficient in perfectly-elaborated fibrin, and is indisposed to coagulate. It is in such that spontaneous hemorrhages are most apt to occur; and it is in such also that wounds are least disposed to unite. That a deficiency of fibrin in the blood will produce this indisposition has recently been proved experimentally by M. Magendie.* Now, in inflammatory conditions of the system, there is a tendency to increase in the quantity of fibrin in the blood, so that its viscidity is increased and its coagulum becomes larger and firmer; and a similar change seems to be produced by the local action of the vessels on the blood circulating through an inflamed part. Although, therefore, inflammation, supervening in a healthy state of the system, may be injurious to the reparative processes, and may prevent their regular occurrence, it seems to

* Leçons sur le Sang.

us quite easy to comprehend that, when the system is below par in the condition required, a slight degree of inflammation may be salutary. We are well aware that whatever explanation of this fact, to which we think that he ought to have alluded, might be given by Dr. Macartney, it would be different from that which we have offered; since *he* does *not* regard the effusion of plastic lymph as *ever* a consequence of inflammation. So far as we have yet advanced, it will be seen that we coincide entirely in the opinions of Professor Burns,* from whom we shall quote a passage that seems to us to contain the essence of the question. "Reunion of parts by the process of adhesion is neither more nor less than the reestablishment of the natural function of nutrition, over surfaces in a state of apposition. It is a natural process, existing, however, in a forced or excited degree, and going on also in a state of progressive perfection. . . . That adhesion may follow inflammation, we have already seen, and shall again notice; but that it is in its ordinary form a species of inflammation, I cannot admit. . . . Even when accompanied with its highest excitement, and attended with its most vivid and highly-marked phenomena, it is not inflammation: as well may we call blushing inflammation, because it is attended by heat and redness; as well may we call heartburn inflammation, because it is attended by sensation; as well may we call pregnancy inflammation, because there the uterine vessels enlarge and deposit new matter. Inflammation is an action productive of a complete change of the natural condition. It is new in its nature, it is connected with an altered texture, it is an action of disease, tending rather to disorganization than preservation; a condition which may, indeed, stop short of destruction, but which also may proceed through every degree of change and injury to utter ruin. The action, on the other hand, attendant on adhesion, is limited in its degree, not merely the most effectual enemy to inflammation, but absolutely incompatible with it; a sure and certain plan for the restoration of the part, and the security of the system."†

With regard to the nature of the process concerned in the effusion of plastic lymph, it appears to us that Dr. Macartney, and those pathologists who profess to be of the school of Hunter, have erred in two opposite extremes. The latter assert that, because coagulable lymph is thrown out from certain surfaces in a state of inflammation—which lymph, by becoming organized, produces adhesion between the surfaces—adhesion is always the result of inflammation: and Dr. Macartney argues, on the other hand, that, because wounds may unite by a medium of organizable lymph, without inflammation, the effusion of that lymph is in no circumstances to be regarded as a consequence of inflammation. This, at least, appears to us to be the drift of his argument; but we must confess that we cannot understand the grounds on which he speaks of this action as a "reputed" consequence of inflammation, rather than a real one. Both parties seem to us to have laid too much stress on the supposed final causes of particular processes. The out-and-out Hunterians maintain that inflammation has an essentially beneficial tendency; and

* Principles of Surgery, vol. i. p. 383. 1831.

† We cannot but feel surprised that these very philosophical views, published several years ago, should have excited so little attention amongst pathologists; and especially that no notice should be taken of them in the article "Adhesion," in the Cyclopædia of Surgery.

that, wherever reparation takes place, it is in consequence of that tendency being called into action. On the other hand, Dr. Macartney assumes, as it appears to us, that the effusion of coagulable lymph has always a reparatory or protective purpose, and that it is *therefore* essentially distinct in character from inflammation, which is a morbid condition, and always injurious. He allows that the adhesions which are formed in consequence of it are frequently injurious, and may even be fatal. But "because evils occasionally result from the adhesion of parts, we are not therefore to conclude that the effusion of lymph is an inflammatory action; or that the union and reorganization of parts are not, in general, salutary." But we must protest against this mode of reasoning upon facts. The question is, are there instances in which the effusion of coagulable lymph occurs without any external excitement, but as a consequence of inflammation, and of inflammation only? We believe that there are but few pathologists who would hesitate in answering this question in the affirmative. We have already expressed our full acquiescence in Dr. M.'s doctrine, that, in the reparation of injuries, the effusion of lymph is to be regarded as a non-inflammatory or physiological process; and we are not altogether disinclined to go further, and to admit that it may, when thrown out under particular conditions on serous surfaces, be regarded as a protective process in which inflammation is alike unconcerned. But here we must stop; since we cannot see that any but the most distorted mode of reasoning can ever demonstrate that the effusion of lymph during spontaneous inflammation of serous membranes, mucous surfaces, cellular tissue, &c. is not as completely a result of that process as the effusion of serum or pus. We have in inflammation of the iris an opportunity of watching its various stages; and it is not unfair to conclude that, where the symptoms correspond, and the tissues concerned are analogous, the process is similar in other membranes whose situation debars them from being inspected during life. In acute inflammation of the iris, every surgeon knows that all the symptoms of inflammation are developed before effusion takes place; and that, just as in the case of the arachnoid, the pleura, or the peritoneum, the effusion may be fibrinous or purulent, according to circumstances, though most frequently the former. The influence of treatment, also, is another important consideration; and it is universally found that, to prevent adhesion in this and other cases, those means are most efficacious which subdue the inflammation. It would be easy to multiply illustrations of this kind; but we do not imagine that many of our readers will be at fault for them. We will only stop to point out that the view we have taken accords better with teleological principles than either of those from which we dissent; since adhesion, when it occurs without inflammation, but as a result of physiological action, may always be regarded as sanative; whilst, if it result from inflammation—a diseased condition—it is as uniformly injurious.

To Dr. Macartney is certainly due the credit of pointing out the *rationale* of an occurrence not previously unknown to practical surgeons, but which had never been referred to any general principle,—namely, the union of open wounds without granulation and suppuration. We shall therefore give his account of this process without abridgment.

"The mode of reparation by the modelling process has never been described; because surgeons, heretofore, did not know that it was possible for open wounds to heal without inflammation in the higher classes of animals. However, when healthy parts are injured, although it may be to the greatest extent, if placed under the most favorable circumstances for carrying on their natural actions, the process of reparation is nearly the same, even in the human subject, as that which I have described as belonging to animals of a simple structure. The pain arising from the injury soon ceases. No tumefaction ensues, separating the edges of the wound, and its surfaces are not only disposed to lie in contact, but even to approach each other so much that they cannot be kept asunder by mechanic restraint: there is, therefore, no necessity for the effusion of lymph; and, as there is no cavity to be filled up, granulations are not formed. The surfaces of the wound, although they come into contact, do not unite by vessels shooting across; they are smooth, red, and moistened with a fluid, which is probably serum, and present the appearance of one of the natural mucous surfaces of the body. If any parts have been killed by the injury, they are separated by simply as much interstitial absorption as is sufficient to set them free. The wound is finally healed by the same means which determine the shape of the natural parts of the body. It gradually diminishes in extent until it is obliterated; or it may be cicatrized before the surfaces are abolished, after which the same process of natural growth goes on, until no part of the original wound is left. The cicatrix which succeeds the cure of injury by the modelling or growing process is small, pliant, free from those callous adhesions to the parts underneath, and the morbid sensations that so often belong to those cicatrices, which have for their bases the deposits of lymph, or the new-formed structures called granulations. When the modelling process, or cure by the natural growth, goes on perfectly, there is no inflammation in the part, and the patients are so entirely free from all uneasy sensations, that I have known instances of their being ignorant of the real site and extent of the injury, until they had examined the part with their hand or saw it with a looking-glass. It might be anticipated that, as this mode of reparation bears so strong a resemblance to the natural formation and development of parts, it is the slowest mode; but this is of little account when compared with its great advantages in being unattended with pain, inflammation, and constitutional sympathy, and leaving behind it the best description of cicatrix. It constitutes the nearest approach, in the higher classes of animals, to that regenerative power which is exhibited by some of the inferior tribes." (pp. 53-4.)

We have said that surgeons have not been altogether ignorant of the possibility of this mode of reparation. It is evidently that which often takes place under a clot of blood, when the constitutional state and the condition of the parts are favorable. In Hunter's treatise we find a section of the chapter on Union by the First Intention devoted to the consideration "of Scabbing;" and, from the description he gives of the process, it is quite evident that his views of it were not really very dissimilar from those of Dr. Macartney.

"Even where the parts are not brought together, so as to admit of union by the first intention, nature is always endeavouring to produce the same effect. The blood which is thrown out in consequence of the accident, and which would have united surfaces brought into contact, is in part allowed to escape, but, by its coagulation on the surface, a portion is there retained, which, drying and forming a scab, becomes an obstacle to suppuration. The inflammation in this case may be greater than where union can be effected, but not nearly so great as when suppuration takes place. This might be considered as the first mode of healing a wound or sore, for it appears to be the natural one, requiring no art; and, in the state of parts before mentioned, the complete union is in some degree indebted to this mode of healing, by uniting the edges that were not or could not be brought into close contact, by means of a scab: proper attention to this has, I believe, been too much neglected. Many wounds ought to be allowed to scab in which this process is now prevented; and this arises, I believe, from the conceit of surgeons, who think themselves possessed of powers superior to nature, and therefore have introduced the practice of making sores of all wounds. . . . . . How far this practice may be extended is not yet ascertained. A small wound doing well under this treatment is a common case, and some examples of large wounds are mentioned, though these do not so generally succeed; but I do not know that there is any danger in the attempt." (*Palmer's Edition of Hunter*, vol. iii. pp. 262-3.)

To the last sentence of this passage, Mr. Palmer subjoins the following illustration:

"Mr. Wardrop, in his Lectures on Surgery, has related a very remarkable case, in which 'the largest wounded surface' he ever beheld, arising from the ablation of a diseased breast, almost entirely healed under a crust of blood, which remained on the surface of the wound for upwards of thirty days; the process of granulation,* approximation of the edges, and cicatrization of the wounds going on underneath, with scarcely any irritation or inflammation of the adjacent integuments."

Artificial coverings of the same kind have been recommended by many practitioners in the cure of ulcers; and the account recently given by Dr. Greenhow† of his success in treating burns, not only superficial but deep, by entirely excluding them from the air, evidently corresponds with that of Dr. Macartney. The plan was suggested to Dr. G. by an accident. A boy fell with both his arms into a kettle of boiling pitch, which glued the sleeves of the jacket so firmly to the arms that the attempt to remove them was abandoned in despair. The hands, which were cleaned and treated in the usual way, suppurated copiously, and sloughs separated; but, a month before they were entirely healed, the jacket sleeves became detached from the arms, and, on their removal, the surface was found perfectly healed, although it was evident that sloughs had come away, and although there had been no suppuration, no offensive smell; nothing, in fact, to indicate that any process was going on. By artificially imitating this coating by means of a resin ointment applied in a liquid state, Dr. G. has been able to obtain similar results in subsequent cases; suppuration never taking place except from accidental causes, and the sloughs, where the parts were deeply injured, peeling off like pieces of shrivelled leather, as the surface skinned beneath them. The advantages of this mode of healing burns are most evident. The system is saved from the constitutional irritation which a large suppurating surface necessarily keeps up, and which so frequently leads to a fatal result; and the cicatrices, if such they are to be called, resemble the natural surface both in appearance and character; and none of those contractions take place of which every practitioner has to regret the occurrence under ordinary modes of treatment with the best management.

We have alluded to these facts more at length than may appear necessary, partly because Dr. Macartney has not taken such notice of them as they seem to demand, and partly to show that the doctrine which he has brought forward on reparation by the "modelling process" is perfectly conformable to the experience of the profession. Dr. Greenhow speaks of this mode of healing as "by the first intention;" which plainly shows that, in his mind, it is distinct from inflammation. We cannot altogether concede to Dr. Macartney, then, the merit of first directing

* Although Mr. Wardrop here speaks of *granulation*, it is evident that he did not *see* it; but that he only supposes the process to have taken place, because he was not aware that open wounds could be filled up in any other way.

† Medical Gazette, October 13, 1838. (See p. 287 of our present Volume.)

attention to this process, since such evident notice of it has been taken by Hunter and by practitioners of the present day. He scarcely alludes, in fact, to the nature of the scabbing process; and leaves us in doubt as to what he considers the mode of union in such cases to be. But we gladly give him the credit of having explained the *rationale* of this mode of reparation, by showing its analogy to the reparative process in the lower classes of animals, in which inflammation is certainly not concerned; and of pointing out the principles of treatment by which this most desirable end may be attained in a great variety of cases. This is one of the grand improvements which are gradually elevating surgical pathology to the rank of a science, and which will gradually render practice a scientific and not an empirical art.

We should be sorry for our readers or for Dr. Macartney to think that in our previous remarks we have been directed by a spirit of depreciation. Our only aim is the establishment of truth; and, whilst none can take greater delight than we do at the success of any attempt to reduce under general principles a chaotic mass of empirical facts, we still feel that these facts are not to be despised; since from them may be deduced valuable rules of treatment, of limited application indeed, but which have still been found capable of leading to a successful result in cases in which they have been judiciously practised. And, as another instance of the same approximation, on a limited scale, to Dr. M.'s general principles, we may advert to Mr. Syme's plan of treating wounds by water-dressing, and such other means as tend to abate instead of arousing inflammation.*

To effect the reparation of injuries by the approximating or modelling process, when direct union is inadmissible, is the object of Dr. Macartney's treatment, to which we shall presently recur; but, to set the nature and advantages of it in a clear light, we shall quote a case which he gives in illustration of its effects compared with those of the usual method.

"Mr. O—y was directing a labourer to drive a stake into the ground with a heavy mallet: the man, mistaking what was said to him, struck down the mallet with great force, before his master's hand was removed from the top of the stake. The blow crushed the forefinger, and laid it open from one end to the other. The gentleman, being acquainted with my mode of treating such injuries, and being in possession of the steam apparatus invented by me, placed his hand under the influence of steam, which speedily removed all pain, and he afterwards applied the water-dressing. The wound went on towards a cure, in the manner I have described under the name of the approximating or modelling process. No granulations were formed, nor pus generated. The edges of the wound came together, and presented the appearance of a red, smooth fissure. He was now told by a surgeon that union would not take place without the aid of adhesive plaster; on which, he consulted me respecting what he should do. I advised him (in order to satisfy him) to try the effect of putting a piece of adhesive plaster around the middle of the finger, and to continue the water-

* It has been insinuated, in a recent review of Dr. Macartney's work, that Mr. Syme's Principles of Surgery "contains the major part of Dr. Macartney's peculiar ideas upon inflammation," taken without acknowledgment from Dr. Macartney's Lectures. We cannot but consider this accusation as altogether unjust, as any one may convince himself who will take the trouble to compare the work in question with the doctrines of Dr. Macartney. Mr. Syme's paper on the union of wounds was published in the year 1825; not so very long, therefore, after Dr. Macartney was appointed to the Dublin professorship. The treatment there recommended is obviously based on the author's experimental results, and not on any general principles like those enunciated by Dr. Macartney.

dressing to the remainder. He followed this direction, and in two days after, the wound opened under the plaster, the skin inflamed round the part, granulations shot up in that portion of the wound, and pus was secreted. The gentleman was now convinced of his error. He returned to the water-dressing, under which the granulations that had formed faded, the secretion of pus declined, the inflammation which had been produced by the plaster subsided, and the wound healed without further trouble in the manner that I had intended. After cicatrization took place, the surfaces of the wound gradually rose to the level of the surrounding skin: he finally recovered the perfect use of his hand, and no appearance of the injury remained except a mere line of white cicatrix, which only possessed hardness at that part where the adhesive plaster had been applied, and a little below it next the palm of the hand. The two extremities of this wound were healed in ten days after the accident; and the middle, to which the plaster was applied, in seven days afterwards." (pp. 212-13.)

The last mode of reparation admitted by Dr. Macartney is that by granulations, the growth of which he regards (very justly, we think,) as not in itself an inflammatory process, but as the means of reparation employed by nature under the unfavorable circumstances of irritation or the continuance of inflammation; proving that parts previously in a healthy state are disposed to heal in despite of many impediments thrown in their way. The following extracts present his view of the nature of the process.

"It would appear that the granulation structure, being endowed with more vascularity and a higher degree of organization than can be acquired in a short time by effused lymph or blood, is the reason of its being formed for the purpose of reparation, when the parts are placed under unfavorable circumstances to accomplish a cure; for we find that, if lymph or blood be shed on a common ulcer by incisions of its surface, and perfectly inclosed so as to avoid all external irritation, and kept in an easy state of sensation [?], it unites with the surfaces of the ulcer, and acquires organization, as when the same substance is shed in common wounds. The existence of granulations has been supposed to be necessary to fill up deficiencies: this, however, is not the correct explanation; for we meet with very considerable vacancies filled with lymph, which is never converted into granulations; as in the cases where recent incised wounds are imperfectly closed, but nevertheless are healed by the first intention. The necessity for the granulative process seems entirely to arise from the parts being in that degree of excitement which is not enough to prevent reparation altogether, but to permit it to be effected by a highly vascular medium. . . . And, further, it is only in the beginning that granulations take the place of the natural structure; for the approximation of the edges of a wound or of an ulcer is accomplished by the interstitial absorption; and, finally, wounds that are healed by the granulative process exhibit no more remains of the new medium employed for union than if lymph had been the substance employed. Granulations, therefore, exist for a special purpose; and, that being effected, they cease to occupy the place of the original structure longer or more than is necessary. The ultimate absorption of the granulations is something like the contracting or approximating action which exists in open wounds that have never inflamed; and it does not take place until the inflammation has subsided in a common wound or ulcer.

"The organic structure of granulations is very peculiar: although easily destroyed by injury or a high degree of inflammation, it is endowed with important vital properties. A deposition of lymph, after being united to an exposed surface, as in an ulcer, or in the interior of an abscess, may assume the granulation structure; but the usual manner in which it is formed is by deposition and organization going on together, as in the growth of the natural parts of the body. In whatever mode granulations are produced, they are composed of a fine cellular membrane, into which blood-vessels proceed from the subjacent tissue. . . . In the indolent ulcer the surface is not granular, but depressed, hard, striated, and is nothing more than very imperfectly organized lymph." (pp. 56-7.)

Now, the state of the case appears to us to be this: In union by the deposition of lymph, the reparative matter is deposited unorganized, but in a state capable of becoming organized, which it speedily does, if the condition of the neighbouring parts be favorable. In the modelling process, no deposition takes place, but the reparative materials are applied to the extension of the original tissues. But when the inflamed state of the adjoining tissues and fluids prevents either one of these processes from taking place in a perfect manner, a new substance is formed, which is not capable of ever becoming sufficiently organized to form part of the general structure, although the properties with which it is endowed have an important temporary object. It is evident that, in one sense, the formation of granulations may be spoken of as an inflammatory process, since it is a result of the abnormal condition of the neighbouring parts; but we think that it may be more fairly represented as a perversion or distortion of the natural reparative processes induced by this condition. All are agreed that, without the presence of a certain amount of inflammation, granulations do not appear; and that this inflammation is unfavorable to the final cicatrization of the wound, when an extension of the cutaneous surface takes place over the newly-formed tissue, few can help acknowledging who reflect upon the diminished vascularity that is always evident in the neighbourhood of the wound, and especially at its edges, while the process of cicatrization is taking place. In fact, as Dr. M. has justly remarked, "when the granular structure is prepared by the cessation of inflammation, the mode of healing bears a strong resemblance to that which takes place when open wounds are cured without inflammation having occurred." (p. 62.)

We have already considered one of the processes regarded by Dr. Macartney as erroneously classed among the consequences of inflammation; namely, the effusion of plastic lymph. We shall now briefly advert to another, ulceration. Every one who has read Hunter's treatise, or who has heard it expounded, (and what well-educated surgeon has not?) must remember that he nominally treats of ulceration as a consequence of "ulcerative inflammation." But a closer examination of his descriptions will show that he entertained a very different notion of the process from that which such a designation might reasonably suggest to the minds of others. He distinctly states that ulceration is produced by an action of the absorbents, resembling that which introduces aliment into the system ; the *disposition to be absorbed* being given, in the cases in question, by a previous state of irritation and inflammation. The following passages will unequivocally show this to be his opinion:

"The dispositions of the two parts of the living body which absorb and are absorbed must be of two kinds respecting the parts, one passive and the other active. The first of these is an irritated state of the part to be absorbed, which renders it unfit to remain under such circumstances, the action excited by this irritation being incompatible with the natural actions and the existence of the parts, whatever these are, which therefore become ready for removal, or yield to it with ease. The second is, the absorbents being stimulated to action by such a state of parts, that both conspire to the same end. . . . . Absorption in consequence of disease is the power of removing complete parts of the body, and is in its operation somewhat similar to the modelling process, [that by which the natural form of the body is preserved, or altered in the stages of its growth,] but very different in the intention, and therefore in its ultimate effects. This process of removing whole parts in consequence of disease, in some cases produces effects which are not similar to one another: one of these is a sore or ulcer, and therefore I call it ulcerative. In other cases no ulcer is produced, although whole parts are removed, and for this I have not been able to find a term; but both may be denominated progressive absorption. . . . The process of the removal of parts of the body, either by interstitial or progressive absorption, answers very material purposes in the machine, without which many local diseases could not be removed, and which, if allowed to remain, would destroy the person. It may be called in such cases the natural surgeon." (*Palmer's edition of Hunter,* vol. iii. pp. 465, 461, 463.)

We believe the fact to be, that Hunter felt encumbered by the term "ulcerative inflammation," the use of which among surgeons of his time was the consequence of their erroneous notion that the breach of substance was occasioned by the dissolution of the tissues into pus; a notion of which Hunter demonstrated the fallacy. It is evident, then, that Dr. Macartney differs but little from Hunter in the character he assigns to ulceration; but we shall again state his opinion in his own words. After pointing out the concurrent agency of the arteries and absorbents in the natural growth of the body, he adverts to the operation of the latter in the removal of extraneous substances and in the imbibition of aliment. He then continues,

"There is no essential difference between the most salutary actions of the absorbents and the most destructive ulcerations: in all cases these vessels are governed by the same law, which is to remove parts that are not necessary or not fit to remain. There is every reason to believe that ulceration always takes place, because the vitality or the organization of the parts have been impaired by inflammation, weakness, pressure, or other external injuries. All new-formed parts and morbid growths are peculiarly subject to ulceration on the slightest provocation, because their vital powers are low, and their structure imperfect: hence cancer, fungous and medullary tumours, are sometimes rapidly destroyed by phagedæna or by slough. The structure in which scrofulous deposit exists is liable, on slight excitement, to set up the ulcerative process. The inferior parts of the body, as they have less vital power than the upper, are most frequently the seat of ulcers. As we see that the process of ulceration, in so many cases, is palpably intended to remove parts which are first rendered incapable of performing natural and healthy actions, we have a right to assume that there is no instance of parts ulcerating whose existence is necessary, and whose structure is quite sound. [In cases where the structure is sound, but the existence unnecessary, we have atrophy, without breach of continuity, produced by interstitial absorption, unbalanced by deposition.] If this view of the nature of ulceration be correct, it is of the greatest practical importance, as it would instruct us to address our remedies to the causes of ulceration, by which the effect may be often rendered unnecessary." (pp. 42-3.)

We do not see that there is much novelty in these remarks, since, as far as our experience extends, intelligent practitioners have long been in the habit of acting upon them; but we gladly concede to Dr. Macartney the credit of having placed the real nature of the ulcerative process in a striking and self-evident light; and, taken in connexion with his doctrines on the subject of reparation, the physiological character (so to speak) of both is, to our minds, satisfactorily made out. Adverting to the designation "natural surgeon," applied by Hunter to the ulcerative process, he remarks,

"I should be disposed to apply the same name both to the effusion of lymph and the process of absorption. The one acts like the surgeon that unites parts, the other like the one who removes them because they are not fit to remain; and it would not appear more justifiable to call adhesion and ulceration inflammatory processes, than to consider the operations of surgeons themselves as particular modes of inflammation." (p. 44.)

The injury to the organization and vital properties of parts, which renders them unfit to remain constituents of the fabric, and occasions them to be thrown off *en masse* by mortification, or to be removed by the ulcerative process, is an undoubted consequence of inflammation.

We shall now briefly advert to the *treatment* recommended by Dr. Macartney for the purpose of giving full effect to the powers of nature in the reparation of injuries, by preventing or subduing the inflammatory action which is so injurious to them. This principally consists in the careful regulation of temperature, and the constant application of moisture. The immediate effects of injuries, especially of such as act severely upon the sentient extremities of the nerves, are, according to him, best abated by the action of *steam* at a high but comfortable temperature, the influence of which is gently stimulant, and at the same time extremely soothing to the feelings of the patient. When the remedy is used immediately after severe injuries, especially those accompanied with a peculiar overcoming pain and a shock to the nervous system, "it removes all pain and consciousness of injury in a very short time." Its stimulant action is seen by its operation on ulcers healing with luxuriant granulations, which it makes tender, painful, and liable to bleed; and it does not, therefore, seem evident why it should have such a soothing influence in wounded surfaces; but its *modus operandi* in improving the condition of indolent ulcers can be easily comprehended. After the pain and sense of injury have passed away, the steam, at a lower temperature, may be continued; and, according to Dr. M., no local application can compete with this when inflammation is of an active character. For assisting the reparative process, however, water-dressing will generally answer sufficiently well; its principal object being the constant production of a moderate degree of cold, which diminishes but does not extinguish sensibility and vascular action, and allows the reparative process to be carried on as in the inferior tribes of animals. The reduction of the heat in an *extreme* degree, as by the application of ice or iced water, is not here called for, and would be positively injurious; since it not only renders the existence of inflammation in the part impossible, but, being a direct sedative to all vital actions, suspends also the process of reparation. Dr. Macartney gives a faithful history of the employment of water as a curative application to wounds from the earliest ages, which he thus concludes:

"It is quite manifest, from the history I have given of the employment of water in its liquid state for the cure of wounds and inflammation, from the earliest period to the present time, that I do not claim the discovery of the remedy; but that I have been the means of introducing it to the attention of the profession in these countries is a matter of too much notoriety to admit of dispute. I have also connected the use of it with general views on the nature of inflammation, which (whether right or wrong) are peculiarly and distinctly my own; and I have demonstrated the possibility of open wounds healing without inflammation, and without the medium of either coagulable lymph or granulations; a fact which, as far as my information extends, has not even been hinted at by any writer on surgery, either ancient or modern." (p. 189.)

We do not think that, on the whole, Dr. Macartney here assumes too much to himself; and he has shown himself so free from the egotistical spirit which possesses some of our claimants to modern discoveries, that we should be doing him injustice did we not accord to him, with the trifling exceptions we have already mentioned, all that he claims regarding the exposition of the *rationale* of the reparative processes, and the enlarged views we have thus obtained as to the application of curative means. We are unwillingly compelled to defer until another occasion the consideration of the subject of inflammation itself, having, in this, our first article, occupied our prescribed space with the discussion of preliminary questions; of which, however, no one will deny the importance. We have not entered into a minute description of Dr. Macartney's mode of treating injuries, partly because we have on a former occasion* called the attention of our readers to the value of cold water in the treatment of injuries; and partly because we consider it but fair towards Dr. M. not to prevent the necessity of reference to his work. This reference we would strongly urge our readers to make; and it must be acknowledged that a condemnation of his treatment will be decidedly unjust until it has been fairly tried, with all the precautions that he recommends. We cannot, perhaps, quite go the whole way with him in asserting that the practice founded on his doctrines "is at present established too extensively, and confirmed by the experience of too many individuals, to admit of controversy;" but we may safely say that, in a great variety of cases, no doubt as to its efficacy can exist in the minds of those who will fairly weigh the evidence in favour of it; and that these cases are of a nature in which efficacious treatment is of the highest importance for the preservation of life or limbs. We more especially allude to severe injuries involving the joints, and gunshot and extensively lacerated wounds. In so far, therefore, as he has been instrumental in thus alleviating human suffering, he well deserves the gratitude not only of the profession, in whose science he has made an important advance, but of mankind at large.

---

### Art. VI.

* British and Foreign Med. Review, Vol. III. p. 114 et seq.

